# Espionage Scandal Leads Science News

**DOI:** 10.1100/tsw.2001.52

**Published:** 2001-05-18

**Authors:** Shauna M. Haley

## Abstract

Two Japanese molecular biologists are charged with espionage in a case that could strain scientific relations between the U.S. and Japan, report both Nature and Science in their top stories this week.

On 10 May, Takashi Okamoto and Hiroaki Serizawa were indicted by the U.S. Justice Department for allegedly stealing DNA constructs and cell lines from the Cleveland Clinic Foundation (CCF) in Ohio and transporting them to the Institute of Physical and Chemical Research (RIKEN) in Japan. Charges were filed by the U.S. federal law enforcement agency under the 1996 Economic Espionage Act. The act was passed mainly to protect against the theft of technology industry trade secrets; however, the invocation of the act in the Okamoto-Serizawa case carries with it the implication that the Japanese government may have benefited from the reagent theft, reports *Nature*. RIKEN's Brain Science Institute president, Masao Ito, is concerned about those implications damaging RIKEN's reputation, says *Science*. The research facilities and programs at RIKEN are comprised of 245 of its own researchers, with additional collaborations throughout the world. RIKEN's Genomic Sciences Center director, Akiyoshi Wada, commented in *Nature* that, "Using words like 'espionage' could ruin any hope of establishing trust between researchers." Japan will have to cooperate with the U.S. to extradite Okamoto — another factor raising the stakes in the diplomatic minefield being laid by the particulars of this case.

The theft occurred in July 1999, according to Justice Department records, while Okamoto, who had lead an Alzheimer's research team at CCF for over two years, was transitioning to a new position at RIKEN. Three other accomplices aided the scheme, according to allegations. Colleague Serizawa, who resides in the U.S., is the only one named in the indictment. The scandal is puzzling for many reasons. First, scientists often take samples of research projects from one job to the next. Okamoto is accused of taking samples without permission and sabotaging other reagents left in his lab. George Stark, who heads the CCF research center where Okamoto worked told *Science* that Okamoto would have been permitted to take samples to RIKEN had he asked. Further, notes the journal report, no patent-worthy work was created in Okamoto's lab. Masao Ito of RIKEN also explained to *Science* that the nature of Okamoto's research had changed from cell signaling and apoptosis studies to investigations of the cholesterol metabolism thought to be central to the formation of plaques in the brains of Alzheimer's sufferers. The stolen materials would not be used in Okamoto's current projects. Curiously, the fate of the missing reagents is unknown and investigations into their whereabouts continues. Serizawa is expected to appear in a Cleveland federal court next week, where more light may be shed on this strange scandal. Whether or not Okamoto will be extradited has not been established.

This Week in Nature and Science, May 18, 2001
© 2001 TheScientificWorld, Inc.
